# Assessing the Impact of a Novel *Trichoderma* sp. Strain STP8 on Lettuce Yield and Mineral Content

**DOI:** 10.3390/jof11100743

**Published:** 2025-10-17

**Authors:** Snježana Topolovec-Pintarić, Martina Stvorić, Božidar Benko, Sanja Slunjski, Neven Matočec, Ivana Kušan

**Affiliations:** 1 Division of Phytomedicine, Department of Plant Pathology, Faculty of Agriculture, University of Zagreb, 10000 Zagreb, Croatia; 2 Department of Phytomedicine, Križevci University of Applied Sciences, 48260 Križevci, Croatia; mstvoric@vguk.hr; 3 Division of Horticulture and Landscape Architecture, Department of Vegetable Crops, Faculty of Agriculture, University of Zagreb, 10000 Zagreb, Croatia; 4 Division of Agroecology, Department of Plant Nutrition, Faculty of Agriculture, University of Zagreb, 10000 Zagreb, Croatia; 5 Laboratory for Biological Diversity, Ruđer Bošković Institute, 10000 Zagreb, Croatia

**Keywords:** agronomic traits, bio-fertilizer, green technology, *Lactuca sativa* L., macronutrients

## Abstract

The fungal genus *Trichoderma* is highly valued in agriculture for its versatile roles, mainly as a biocontrol agent against plant pathogens. Recently, its use as a natural biofertilizer has gained attention, as *Trichoderma* spp. promotes crop growth and improves yield by enhancing the rhizosphere environment and activating plant defences. Globally, over 250 *Trichoderma*-based products dominate 60–90% of the market, but their efficacy can decline during transportation and storage. Additionally, concerns about their impact on native soil biodiversity have led to interest in using locally adapted, native strains. The novel native strain of *Trichoderma* sp. STP8 (formerly *T. koningiopsis* agg. STP8) previously showed strong antagonism against *Sclerotinia sclerotiorum* and promoted lettuce growth in greenhouse conditions. This study evaluated *Trichoderma* sp. STP8’s effectiveness in field-grown lettuce, revealing yield increases of 16.6% to 30.5%. The most significant gains occurred when *Trichoderma* sp. STP8 was applied before head formation, 26 days after planting. That was in one treatment with two applications (at seedling planting and after 26 days) and another with three applications (at sowing, at seedling planting, and after 26 days). These results demonstrate *Trichoderma* sp. STP8’s potential as a sustainable biocontrol and biofertilizer agent for lettuce, encouraging further research across different agricultural systems, including hydroponics and soil-less medium.

## 1. Introduction

Lettuce (*Lactuca sativa* L., Asteraceae) is a popular leafy vegetable worldwide, cultivated on approximately 1,260,000 hectares with a total yearly production of around 28 million tons (alongside with chicory). According to official data, in Europe it is cultivated on 127,311 hectares, producing about 3.3 million tons per year [1]. In Croatia, it is grown on 300 hectares of open fields and 64 hectares in greenhouses, with an annual total production of 6932 tons (1650 t in greenhouse) [2]. Lettuce is recognized as an important functional food due to its low calories, fat, and sodium content, while being rich in vitamins, folate, dietary fibres, and essential minerals such as phosphorus (P), calcium (Ca), magnesium (Mg), and iron (Fe) [3,4]. Head lettuce contains on average about 94–96% water, along with 2.0–2.3% sugars, 0.5–0.6% crude cellulose, 0.4–0.6% mineral substances, and 1.0–1.3% crude protein [5]. Its leaves are particularly high in vitamins C, E, and B complex, and it is a good source of potassium (K) while containing low sodium levels (Na). The content of minerals varies among different lettuce types and is influenced by soil conditions. Additionally, lettuce contains bioactive compounds such as polyphenols, carotenoids, and chlorophyll, which offer various health benefits [6].

The growth cycle of lettuce is highly dependent on temperature, with crops typically reaching maximum development during the warmer part of a year in about 45–60 days after transplanting, depending on the variety. In contrast, winter-grown lettuce may take between 90 and 120 days until harvest. Elevated temperatures, combined with high light intensity and longer daylight hours, accelerate growth by promoting faster leaf development, resulting in broader leaves and quicker head formation [5]. Due to its short growth cycle, it is possible to cultivate 2–3 crops of lettuce per year.

Fungi from the genus *Trichoderma* (Hypocreales, Ascomycota) are rhizosphere inhabitants, the members of soil and plant mycobiota, playing a crucial ecological role in both natural and agricultural ecosystems, including the bioremediation of polluted environments [7]. As one of a dominant component of rhizosphere microbiota across virtually all terrestrial ecosystems, *Trichoderma* spp. can enhance overall plant health by creating a favourable rhizosphere environment that supports nutrient availability and suppresses pathogens. Their agricultural importance primarily lies in their strong antagonism against various soil-borne plant pathogenic fungi, which has driven their commercial success as bio-fungicides, typically applied as soil treatments and seed coatings [8,9,10]. But some *Trichoderma* spp. colonize the root surface, even penetrating the epidermis and a few cell layers below in the root tissue, demonstrating their high opportunistic potential by establishing a mycorrhizal relationship with the plant host [11,12]. Due to that, *Trichoderma* spp. can alleviate extrinsic as well as intrinsic stresses and activate plants’ natural defence mechanisms to preserve the health of cultivated plants, which, as new studies have shown, can be heritable [7,13,14,15]. Also, *Trichoderma* spp. may cause significant biochemical changes in plant contents of carbohydrates, amino acids, organic acids, and lipids improving dry weight biomass [16]. In addition, their potential to improve water and nutrient uptake contributes to increased plant height, root and shoot growth, and faster seed germination as well as sprouting. Owing to this fertilization potential, *Trichoderma* spp. has gained even greater agricultural utilization for green technologies, marketed as plant inoculants or plant strengthening agents, and biofertilizers [17,18].

Beside the well-known positive effects of *Trichoderma* spp. application on soil-grown crops, recent research also confirmed positive effects on lettuce growth, yield, and mineral accumulation in hydroponic [19,20,21] and aquaponic growing systems [22]. Application of *Trichoderma* spp. in these systems also enhances the efficiency of mineral fertilizers and helps reduce the amounts required.

The availability and distribution of *Trichoderma*-based biofertilizers are more widespread than commonly recognized and are expanding, partly because they are easier to register, since they are not classified as pesticides, and face less regulatory pressure to reduce chemical pesticide use [23]. Due to the shortened life span of the *Trichoderma* product during transportation, storage, and application, a possible weakened biological control effect may occur after application in the field [10,24]. Moreover, the negative impact of *Trichoderma* species from bioproducts on the biodiversity of not only native *Trichoderma* species but also other organisms (e.g., plants, bacteria, other fungi) is receiving more attention in research studies [7,23,25,26,27]. Therefore, the application of an autochthonous *Trichoderma* sp. strain is possibly more appropriate than different commercial *Trichoderma* spp. products containing an allochthones strain.

A stimulant effect of *Trichoderma* spp. was noted in the greenhouse experiments with lettuce [28,29,30,31,32]. Yet, there is still limited research on its beneficial effects when used in open-field lettuce cultivation. Our recent greenhouse study also demonstrated the strong biofungicidal potential of a native Croatian strain, a novel strain of *Trichoderma* sp. strain STP8 (previously as *T. koningiopsis* agg. STP8), against *Sclerotinia sclerotiorum* in association with lettuce plants, as well as biostimulant effects that resulted in increased lettuce yields [33]. The obtained results led to the setup of a field trial with the aim of investigating the potential of the *Trichoderma* sp. STP8 strain as a lettuce yield promoter and its influence on mineral content. The hypothesis posited that the *Trichoderma* sp. STP8 strain could enhance growth parameters without compromising plant quality.

## 2. Materials and Methods

### 2.1. Isolation and Identification of the Trichoderma sp. Strain

In this study, the novel fungal strain *Trichoderma* sp. STP8 was utilized. The isolate was obtained from humus soil at the experimental vegetable garden site and was originally isolated from the lettuce roots infected with *Sclerotinia sclerotiorum* [33]. The axenic culture was maintained on potato dextrose agar (PDA) media and stored in the temporary laboratory collection under the code STP8 (*Trichoderma* sp.). The strain was taxonomically determined by molecular methods, and the process of DNA isolation, amplification of ITS, *rpb2* and *tef*1 gene regions, and sequencing is presented in detail in the previous work [33].

The Basic Local Alignment Search Tool (BLAST, https://blast.ncbi.nlm.nih.gov, accessed on 6 October 2025) was used for searching similar sequences in GenBank. Sequence alignment of the dataset was achieved individually on ITS, *rpb2* and *tef1* using MAFFT vers. 7.490 [34,35], available as the Geneious Prime plugin (Geneious Prime 2025.0.3 Build 2024-11-22 (Biometers, Oakland, New Zealand)). The ITS dataset was not used in the further phylogenetic analysis due to the weak barcode potential of this DNA region in the genus *Trichoderma* [36]. The best substitution model was selected by Smart Model Selection in the PhyML online resource (http://www.atgc-montpellier.fr/phyml/, accessed on 7 October 2025) [37], TN93 for the *rpb*2 dataset and HKY85 for the *tef*1 dataset, respectively. All species included in the phylogenetic analysis are listed in Table 1, with *Protocrea farinosa* designated as the outgroup [38]. After being aligned and trimmed, concatenation of *rpb*2 and *tef*1 alignments was accomplished using Geneious Prime 2025.0.2. The concatenated alignment contained 1400 character positions including gaps, with 819 character positions for *rpb*2 and 584 characters positions for *tef*1. Phylogenetic analysis of concatenated *rpb*2 and *tef*1 datasets was conducted using maximum likelihood (ML) in PhyML 3.3.20180621, available as the Geneious Prime plugin [39], by applying the bootstrap approximation with 1000 replicates. Bayesian inference (BI) analysis was performed in MrBayes 3.2.6, available as the Geneious Prime plugin [40] under the GTR + G substitution model. BI analysis was executed for 6,000,000 generations, sampling trees and other parameters every 1500 generations. The default numbers of chains (four) and heating parameters were used. Posterior probabilities (BPPs) were calculated after discarding the first 1000 sampled trees. The phylogenetic trees were visualized and annotated using FigTree vers. 1.4.3 (http://tree.bio.ed.ac.uk/software/figtree/, accessed on 13 October 2025).

### 2.2. Field Trial

The field trial was conducted at the Maksimir experimental field (45°49′ N, 16°02′ E), Department of Vegetable Crops, Faculty of Agriculture in Zagreb, Croatia. Prior to the experiment, comprehensive soil analysis was performed (Table 2), revealing a neutral soil pH (pH H_2_O: 7.5; nKCl: 6.86), low humus content (2.22%), but sufficient nitrogen (0.2% N) and potassium (25.5 mg K_2_O·100 g^−1^) levels, while phosphorus was abundant (41.1 mg P_2_O_5_·100 g^−1^). The chemical properties were assessed on air-dried, ground, and homogenized soil samples using standardized methods: soil pH was measured electrometrically with a combined pH electrode in a 1:2.5 soil-to-water suspension, humus content was determined by the Tyurin method, potassium and phosphorus levels were analyzed via the Egner–Riehm–Domingo method [52], and nitrogen content was assessed using the Kjeldahl method [53].

Based on available meteorological data from the Maksimir meteorological station, from May to July, the average monthly air temperature ranged between 15.9 and 21.2 °C, which are optimal for outdoor lettuce cultivation. Rainy days occurred in May and June, totaling 13 days each, and 11 days in July. The average monthly precipitation was 136.1 mm in May, 57.7 mm in June, and 145.7 mm in July [54].

Commercial natural/untreated seeds of Batavia type lettuce cv. Bataille (Nunhems Netherlands BV, Nunhem, Netherlands) were sown on 24 April 2023 into a polystyrene tray with 104 sites, each filled with 32 mL of autoclaved potting mixture substrate Potground H (Klasmann-Deilmann, Geeste, Germany). One seed was placed per hole, and then 0.5 mL of the *Trichoderma* sp. STP8 spore suspension at a concentration of 4 × 10^6^ spores mL^−1^ was applied using a micropipette (Eppendorf Research, Stevenage, UK). Seeds that did not receive this treatment served as controls and for variants where the treatment was omitted.

Seedlings with 3–4 true leaves were manually transplanted at the open field on 25 May 2023, with an intra-row and between-row spacing of 30 cm, resulting in a plant density of approximately 11.1 plants per square meter.

The trial was set up using a randomized complete block design with five replicates, each containing 20 plants, totaling eight treatment variants as shown in Table 3. The *Trichoderma* sp. STP8 spore suspension, at a concentration of 4 × 10^6^ spore mL^−1^ was applied by pouring the suspension directly along the root collar into the soil using a knapsack sprayer (Santaj plastika, Valpovo, Croatia).

Due to the adequate nutrient supply in the soil, fertilization was not applied during soil preparation or lettuce cultivation. The plants were harvested on 7 July, 43 days after planting, and the following morphological parameters were measured: head weight, diameter, and leaf length and width.

### 2.3. Mineral Content Determination

Determination of the plant material mineral content was carried out in the laboratory of the Department of Plant Nutrition, Faculty of Agriculture. Dry matter (DM) is determined by the gravimetric method, where the total dry matter is expressed as a percentage (%) and constitutes the entire amount of matter from the product that does not evaporate under certain conditions. It is carried out in a heated dryer at a temperature of 105 °C. The method is intended for determining the total dry matter in fruit and vegetable products. It is calculated according to the following equation:

(1) Dry matter (%) = (m2 − m0)/(m1 − m0) × 100

where m0 represents the mass of the container and auxiliary material (quartz sand, glass rod and lid) (g); m1 is the mass of the container with the tested sample before drying (g) and m2 is the mass of the container with the residue after drying (g).

The amount of macroelements is expressed as a percentage of dry matter (% DM) for N, P, K, Ca, and Mg, regarding the variable water content of collected samples. Total nitrogen in plant material is determined by the Kjeldahl method. Dried plant material samples were digested with concentrated HNO_3_ by Ethos Up Microwave Digestion System(MILESTONE1200MegaMicrowave Digester, Milestone Srl, Sorisole, Italy). After digestion, the phosphorus content was determined spectrophotometrically, and potassium was determined by flame photometer, while calcium and magnesium were analyzed using an atomic absorption spectrophotometer (AAS) (Thermo Fisher Scientific, Ahaus, Germany) [55].

To convert phosphorus and potassium from their oxide form (P_2_O_5_ and K_2_O) to their elemental form, the following equations were used:

(2) P = P_2_O_5_ × 0.436

(3) K = K_2_O × 0.830.

### 2.4. Assessment of Antagonism on Agar Culture Plates

To evaluate how the *Trichoderma* sp. strain STP8 competes with native soil phytopathogenic fungi in situ, *Alternaria solani* Sorauer, *Fusarium culmorum* (W.G. Sm.) Sacc, *F. solani* (Mart.) Sacc., and *Sclerotinia sclerotiorum* (Lib.) de Bary were isolated from soil where the lettuce was planted. Soil fungi were isolated by using the serial dilution plating technique as described by Shivanand et al. [56]. The antagonistic effects of *Trichoderma* sp. STP8 on the growth inhibition of *A. solani, F. culmorum, F. solani*, and *S. sclerotiorum* were investigated according to Porras et al. [57] using the dual-culture method. The fungal cultures were grown on PDA (Biolife, Milan, Italy) at 21 °C in 90 mm diameter Petri dishes. Mycelial discs with a diameter of 6 mm were removed from the edge of the seven-day-old cultures and transferred to 90 mm diameter Petri dishes containing PDA to form dual cultures. A mycelial disc of pathogen was placed on one side of a PDA plate, while a disc of *Trichoderma* sp. STP8 was placed on the opposite side. For the control plates, a sterile agar disc was used instead of the *Trichoderma* sp. STP8 mycelial disc. Each treatment was carried out in five plates with three replicates (N = 60). The dishes were sealed with paraffin tape (Parafilm, Brand GMBH + CO KG, Wertheim, Germany) and incubated in the dark at 20 °C ± 1 °C for seven days.

On the seventh day, radial fungal colony growth in the direction of the opposite colony was measured manually using a ruler. The maximum and minimum radial growth was measured, and the average radial growth was calculated. The average radial growth was used to calculate the inhibition index (I %) as follows:

(4) I (%) = ((C − T)/C) × 100

where I is the inhibition percentage; C is the radial growth of pathogen (mm) alone (control); T is the radial growth of pathogen (mm) in the presence of *Trichoderma* sp. STP8 [58]. An index value of 50% or more is considered as excellent performance.

### 2.5. Statistical Analysis

Statistical analysis of the achieved results on morphological properties and mineral content was performed in SAS^®^ Software v.9.4 [59]. The differences between the experimental treatments were analyzed by analysis of variance (ANOVA). Determined differences between the average values were compared by the LSD *t*-test and Duncan’s multiple-range test at significance levels *p* ≤ 0.05 and *p* ≤ 0.01.

## 3. Results

### 3.1. The Trichoderma sp. Strain Identification

**Figure 1:**
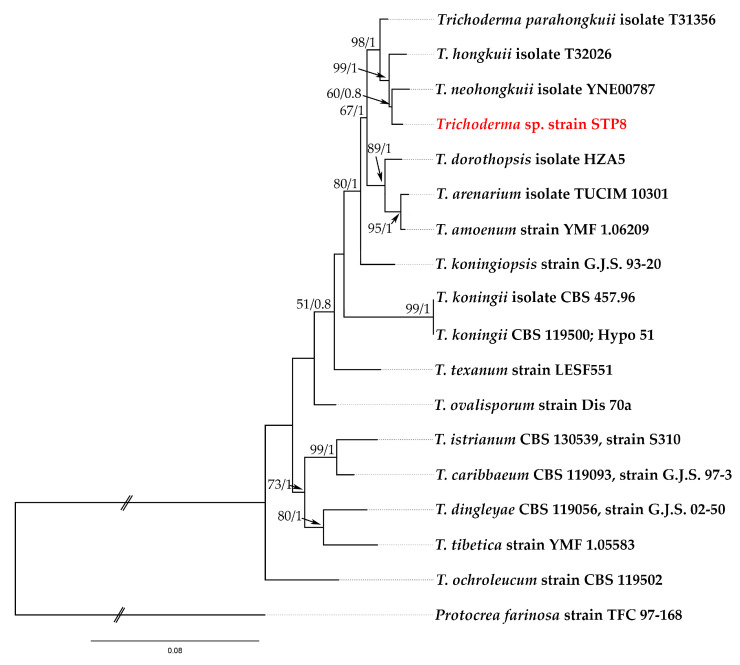
Phylogenetic tree generated by maximum likelihood (ML) analysis using the concatenated sequences of the *rpb2* and *tef1* loci of the *Trichoderma* sp. species clustered in a Koningii clade. Notes are marked with maximum likelihood bootstrap proportions ≥50% (left) and Bayesian inference posterior probability values ≥0.8 (right) (MLBP/BIPP). *Protocrea farinosa* was used as an outgroup. Croatian strain *Trichoderma* sp. STP8 is marked in red color.

In this study, a novel native strain *Trichoderma* sp. STP8, originated from lettuce roots. To identify *Trichoderma* sp. strain STP8 at the species rank, we followed the guidelines of Cai i Druzhinina [36] using three DNA barcode sequence data (ITS, *rpb*2, and *tef*1 gene regions). Megablast search of the NCBIs GenBank nucleotide database using the *rpb*2 and *tef*1 sequences of *Trichoderma* sp. strain STP8 showed that several closest hits belong to *T. hongkuii* C. L. Zhang, *T. neohongkuii* C. L. Zhang and *T. parahongkuii* C. L. Zhang. Phylogenetic analysis of *Trichoderma* sp. STP8 and closely related taxa confirmed clear affiliation of *Trichoderma* sp. STP8 with Koningii clade (Figure 1). Comparison of *Trichoderma* sp. STP8 sequence data to the reference (type) strains of *T. hongkuii*, *T. neohongkuii* and *T. parahongkuii* resulted in 100% identity in *rpb*2 with *T. hongkuii*, and ≥99% to *T. neohongkuii* and *T. parahongkuii* (Table 4). Since *rpb2* is solely not efficient in molecular species recognition of a number of *Trichoderma* spp., e.g., *T. caribbaeum*, *T. istrianum* and others (www.trichoderma.info, accessed on 9 October 2025), we used in our analysis also of the *tef*1 DNA region to ascertain the best possible taxonomic position for the *Trichoderma* sp. STP8 strain. All three aforementioned closely related species were ≤97% similar in the *tef*1 DNA region to the *Trichoderma* sp. STP8 strain (Table 5). Concatenate phylogenetic analysis of both *rpb*2 and *tef*1 datasets showed the strong support of the *Trichoderma* sp. strain STP8 to the *T. hongkuii* species group, with *T. neohongkuii* as a sister species (MLBP = 60; BIPP = 0.8) (Figure 1). Detailed phylogenetic and taxonomic analyses that will include all phenetic datasets are still in progress.

### 3.2. Lettuce Phenotypic Characteristics

In this study, the lettuce cultivar Bataille responded well to the inoculation of *Trichoderma* sp. STP8 in terms of biomass accumulation and marketable yield. In all variants of *Trichoderma* sp. STP8 application, treated plants showed the best quality in terms of morphological parameters as the head weight, diameter, and leaf width were significantly increased in relation to the control. The greatest effect was achieved by applying the *Trichoderma* sp. STP8 suspension twice or three times. The best effect on head weight was achieved in treatment B2 followed by treatment A3. The best effect on head diameter was achieved in treatment A3, A2 and B2. The increment of leaf width was greatest in treatment A1. Although the treated plants showed an increment of leaf length, it was not statistically significant (Table 6).

The *Trichoderma* sp. strain STP8 enhanced growth parameters without compromising plant quality as the hypothesis posited.

### 3.3. Lettuce Mineral Content

**Figure 2:**
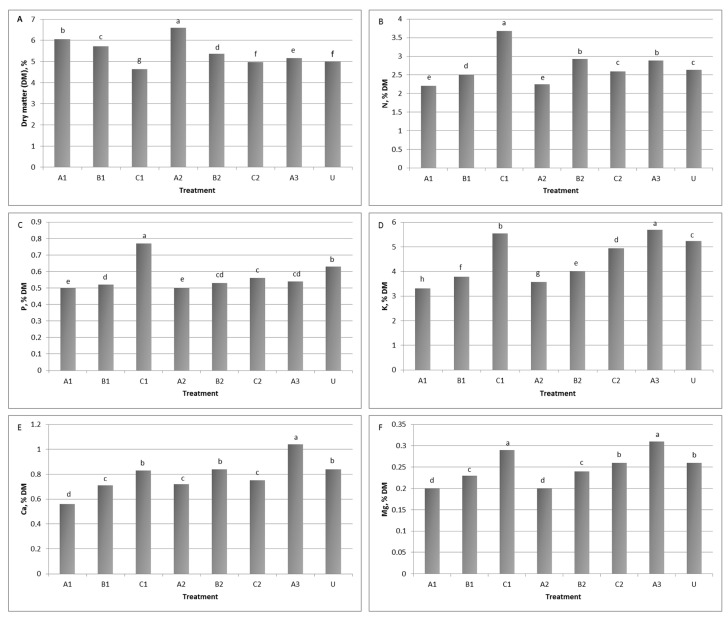
Effect of *Trichoderma* sp. strain STP8 on the (**A**) dry matter (DM) content and mineral content of lettuce leaves’ (**B**) nitrogen, (**C**) phosphorus, (**D**) potassium, (**E**) calcium, (**F**) magnesium. The columns with different letters differ significantly at a significance level of *p* ≤ 0.05.

The application of *Trichoderma* sp. STP8 increased the dry matter biomass and mineral content of lettuce leaves (Figure 2A–F) compared to the untreated control, but also with significant differences between treatments (i.e., time and number of treatments). The significantly highest DM content (6.60%) was determined in treatments A2 and A1, when plants were treated at sowing and planting. In contrast, these plants had the lowest nitrogen, phosphorus, and magnesium contents (2.24, 0.50 and 0.20% DM), even lower than control plants (Figure 2A). Nitrogen and phosphorus contents were highest in treatment C1, when plants were treated only once, 26 days after planting. The content was 20.4 to 40.1% higher than in other treatments, and 28.3% higher than in the control for nitrogen, and 27.3 to 5.1% and 18.2% for phosphorus, respectively (Figure 2B,C). Multiple *Trichoderma* sp. STP8 applications positively affected potassium, calcium, and magnesium contents in lettuce leaves, so the highest values of these minerals were recorded in plants that were treated three times (treatment A3). When compared to the other treatments, contents were higher between 2.5 and 41.8% for potassium, from 19.2 to 46.2% for calcium, and from 6.5 to 35.5% for magnesium. Regarding control plants, the values were 7.9, 19.2, and 16.1% higher (Figure 2D–F).

### 3.4. Antagonism on Agar Culture Plates

All plants in this field trial remained healthy, without any disease symptoms, even though no protective products were used, while laboratory analysis of soil showed the presence of pathogens *A. solani, F. culmorum, F. solani,* and *S. sclerotiorum*.

In vitro tests showed excellent antagonisms of the selected native strain *Trichoderma* sp. STP8 to tested pathogens found on the trial site, through competitiveness and mycoparasitism. *Trichoderma* sp. STP8 grew faster and occupied the largest part of the Petri dish in seven days, and inhibited the growth and parasitized colonies of pathogenic species, except for *F. culmorum*, in the following percentages: *A. solani*—79%; *F. culmorum*—44%; *F. solani*—75%; and *S. sclerotiorum*—77% (Table 7).

### 3.5. Limitation Subsection

The obtained results are based on a field trial conducted at a single location, utilizing the lettuce cultivar Bataille as the sole crop species, over the course of one growing season, which might restrict broader generalization.

## 4. Discussion

Although the genus *Trichoderma* is well-known for its antagonism against phytopathogenic fungi and its beneficial effects on plant growth, these abilities are not uniform across all *Trichoderma* species or even biotypes of the same species, with some exhibiting minimal or no activity, primarily due to genetic differences among strains. Recent taxonomic advancements have revealed biogeographic biases and substrate preferences among species [36], and diversity studies highlight the ecological specialization of *Trichoderma* spp., showing that distribution is influenced by microclimate, substrate types, and complex ecological interactions [60,61,62]. Furthermore, the metabolic products vary among strains, often exhibiting selectivity toward specific plant species or varieties [62,63], and the effectiveness of a strain in promoting plant growth depends on factors such as application method, soil composition, environmental conditions, crop rotation history, and plant interactions [63,64,65]. Consequently, employing autochthonous, locally adapted *Trichoderma* spp. strains may produce more effective results in specific agricultural systems.

In this study, the use of the novel native *Trichoderma* sp. STP8 strain, originating from lettuce roots collected at the same location where the trial was conducted, resulted in an increased marketable yield of the lettuce variety Bataille, contributing positively to similar research [29]. The significant yield increase within just over two months is particularly noteworthy, as lettuce leaves are the final commercial product. The greatest increases in head weight and diameter were observed when the *Trichoderma* sp. STP8 strain was applied before head formation, which in this study occurred 26 DAP: 1) treatment B2—*Trichoderma* sp. STP8 applied twice, at seedling planting and again at 26 DAP; and 2) treatment A3—*Trichoderma* sp. STP8 applied three times, at sowing, seedling planting and 26 DAP.

The observed yield increases of 16.6 to 30.5% align with the findings of Senger et al. [31], who reported yield improvements of 16 to 22% compared to untreated controls, depending on the lettuce variety and *Trichoderma* sp. inoculation dose. Additionally, Lima et al. [30] observed a 45% increase in the fresh mass of the aerial part with *T. virens* and a 15% increase in plant height with *T. koningiopsis*. It should be emphasized that those studies were conducted in optimal/controlled greenhouse conditions. Similar results in lettuce growth promotion were achieved in a study where *T. virens* were applied, resulting in a yield increase of 45% to 67% [66].

The dry weight content significantly differed from the control with *Trichoderma* sp. STP8 application; notably, plants treated once at 26 DAP (treatment C1), showed a 7% reduction in dry matter content. In contrast, other treatments resulted in increases ranging from 3.3% to 24.2%, with the highest increase observed in treatment A2, where *Trichoderma* sp. STP8 was applied twice, at sowing and seedling planting. Similar findings were reported by Senger et al. [31], who observed dry matter content increases between 17.2% and 26.7% across different lettuce varieties in greenhouse conditions. Additionally, our results are comparable to those of Gutiérrez-Chávez et al. [21], although their study was conducted in a floating system, demonstrating that *Trichoderma* spp. application reliably enhances the dry matter content of lettuce regardless of the cultivation approach. These findings suggest the broad applicability of *Trichoderma* spp. across different cultivation systems, which could be valuable for growers seeking effective biological methods to improve yield.

It was determined that *Trichoderma* spp. in soil affects root morphology and architecture, and stimulates root growth, which consequently contributes to nutrient uptake, particularly nitrate, Ca, Mg, and K [66]. The significant *Trichoderma* sp. STP8 interaction with the following elements was observed in determined macronutrient contents: nitrogen, phosphorus, potassium, calcium, and magnesium. Achieved results on mineral content in lettuce leaves corroborate findings from Fiorentino et al. [67], where the use of *Trichoderma* spp. strains led to a notable increase in P, K, and Ca concentrations in lettuce. The increased phosphorous concentrations could be a result of higher phosphorus availability in native soil, induced by *Trichoderma* spp. producing acids (coumaric, glucuronic and citric acid) [68]. However, these results differ from the study by Gutiérrez-Chávez et al. [21], which found that lettuce grown in a floating system had the highest levels of K, Ca, and Mg in control plants, while the nutrient content in plants treated with *Trichoderma* spp. was similar or significantly lower. These findings confirm that endophytic *Trichoderma* spp. can enhance plant uptake efficiency, particularly for nitrogen. Nitrogen can positively impact lettuce by promoting increased growth, yield, and nutrient content [69,70,71]. However, excessive nitrogen availability may diminish lettuce quality by encouraging nitrate accumulation in the aboveground tissues [29]. Importantly, using *Trichoderma* spp. can reduce reliance on external nitrogen sources, including mineral fertilizers and organic amendments like manure and compost, contributing to more sustainable cultivation practices. When comparing lettuce grown under mineral fertilization with those under organic fertilization, de Lima et al. [72] determined a lower dry matter content in organic lettuce. Also, in organic lettuce there were lower levels of magnesium and phosphorus, but higher levels of calcium and potassium.

Species of *Trichoderma* are strong plant invaders capable of colonizing a plant internally in an endophytic manner, allowing them to directly influence plant physiology [17]. This intimate *Trichoderma*–plant relationship induces localized and systemic resistance plant responses to pathogen attack, promoting plant growth and supporting biocontrol. Furthermore, genetic analyses have shown that most of *Trichoderma* spp. biocontrol activity is mediated through their ability to induce plant defence mechanisms, described as systemic disease resistance. Also, analysis has shown that much or most of the biocontrol activity of these fungi is through their abilities to induce plant systemic disease resistance, and that antagonistic mechanisms, antibiosis and mycoparasitism, were found to be due solely to induced resistance [16,17]. In our research, *Trichoderma* sp. strain STP8 was very effective in tests in vitro against soil-borne *A. solani, F. solani* and *S. sclerotiorum*, with an inhibition index between 73 and 79%. As a true mycoparasite of *S. sclerotiorum*, *Trichoderma* sp. STP8 was confirmed in a previous laboratory and greenhouse trial in vivo with lettuce [33]. It should be emphasized that strain *Trichoderma* sp. STP8 was isolated from soil at the experimental field and was originally isolated from the lettuce roots infected with *S. sclerotiorum*. This is in accordance with the conclusion of previous studies that the best results are achieved when the used *Trichoderma* species is isolated from the local areas of the plant and soil [62,63,73]. Moreover, this finding supports the previously postulated idea that the diverse capabilities of *Trichoderma* spp. are encoded within its genome, like the observed genome coevolution in numerous plant–pathogen interactions [68]. Consequently, *Trichoderma spp.* is expected to be effective in disease control under specific conditions of temperature, moisture, and nutrient availability. The hybridization of different strains or species of *Trichoderma* to enhance their beneficial traits has been proposed and commercial mixtures are available [8,68]. Also, in development are other types of consortia, mixtures of *Trichoderma* spp. strains with other organisms, that are known as bioagents [74,75]. Based on this, we will focus our future research at this experimental location on identifying native *Trichoderma* species and their capabilities as biostimulants/biofertilizers, and/or biocontrol agents.

## 5. Conclusions

The positive effects of *Trichoderma* spp. on plant growth depend on the use of an effective *Trichoderma* sp. strain, as the mechanism of its action is influenced by its genetic variability; the method of its application on the seed or root and soil; the genetic variability of the treated plant species and their interactions. Consequently, the outcomes can be unpredictable if the aforementioned factors are uncontrolled, potentially resulting in either positive or negative effects. Notably, the ability of *Trichoderma* spp. to promote growth in short-lived plants like lettuce is particularly remarkable and valuable, offering an eco-friendly approach that requires less financial input by reducing the use of mineral fertilizers, thereby minimizing environmental and health risks while still maintaining crop yields. In this field study conducted at a single location, from where the *Trichoderma* sp. STP8 strain originated, the lettuce cultivar Bataille responded positively to *Trichoderma* sp. STP8 inoculation, demonstrating increased biomass accumulation and marketable yield in just one short growing season. Across all *Trichoderma* sp. STP8 application variants, treated plants exhibited superior morphological parameters, with significant increases in head weight, diameter, and leaf size compared to controls. Additionally, *Trichoderma* sp. STP8 improved dry matter and mineral content in the lettuce leaves. In vitro tests further confirmed the strain’s efficacy, showing strong antagonistic activity against pathogens present at the trial site through mechanisms of competitiveness and mycoparasitism. This native strain was shown to be a promising biostimulant and biocontrol agent. For future research, we plan to investigate the long-term effects of *Trichoderma* sp. STP8 application on lettuce yield over multiple growing seasons, as well as its impact on quality and nutritional content. Finally, we will assess the economic feasibility and sustainability of using *Trichoderma* sp. STP8 in different agricultural conditions, such as lettuce growing in hydroponic and soil-less media, as this can facilitate its broader adoption in practical farming.

## Figures and Tables

**Table 1:** List of sequences included in the phylogenetic analysis.

Species	Reference Culture/Strain/Voucher	*rpb*2	*tef*1	Reference
*Protocrea farinosa*	TFC 97-168	EU703941	EU703896	[38]
*Trichoderma koningii*	CBS 457.96 ^T^	-	AF456909	[41]
*T. koningii*	CBS 119500; Hypo 51	FJ860541	KC285594	[42,43]
*T. amoenum*	YMF 1.06209 ^T^	MT052192	MT070146	[44]
*T. arenarium*	TUCIM 10301 ^T^	MT242310	MT242303	[45]
*T. caribbaeum*	CBS 119093 ^T^; strain G.J.S. 97-3 ^T^	KJ665246	KJ665443	[46]
*T. dingleyae*	CBS 119056 ^T^; strain G.J.S. 02-50 ^T^	KJ665257	KJ665467	[46,47]
*T. dorothopsis*	HZA5 ^T^	MH647795	MK850827	[48]
*T. hongkuii*	T32026 ^T^	OR779477	OR779504	[49]
*T. istrianum*	CBS 130539 ^T^; strain S310 ^T^	KJ665281	KJ665523	[46]
*T. koningiopsis*	G.J.S. 93-20 ^T^	EU241506	DQ284966	[47]
*T. neohongkuii*	YNE00787 ^T^	OR779481	OR779508	[49]
*T. ochroleucum*	CBS 119502 ^T^	FJ860556	FJ860659	[42]
*T. ovalisporum*	DIS 70a ^T^ = CBS 113299 ^T^	FJ442742	AY376037	[50]
*T. parahongkuii*	T31356 ^T^	OR779476	OR779503	[49]
*T.* sp. STP8	STP8	PQ867587	PQ867588	[33]
*T. texanum*	LESF 551 ^T^	KT278920	KT278988	[51]
*T. tibetica*	YMF 1.05583 ^T^	MK779178	MK779179	[44]

Columns *rpb2* and *tef1* indicate the Genbank accession numbers of the respective sequences. ^T^ denotes type (reference) culture, strain or voucher.

**Table 2:** Results of soil chemical analysis at the Maksimir experimental field.

pH H_2_O	pH nKCl	Humus, %	Nitrogen, %	mg P_2_O_5_·100 g^−1^	mg K_2_O·100 g^−1^
7.50	6.86	2.22	0.20	41.1	25.5

**Table 3:** Experimental variants of *Trichoderma* sp. strain STP8 suspension application.

Number of Applications	Variant Mark	Application Time
*Trichoderma* sp. STP8 applied once	A1	at seed sowing
	B1	at planting of seedlings from untreated seeds
	C1	26 days after planting (DAP) seedlings from untreated seeds
*Trichoderma* sp. STP8 applied twice	A2	at seed sowing and at planting of seedlings
	B2	at planting of seedlings (from untreated seeds) and 26 DAP
	C2	at seed sowing and 26 DAP
*Trichoderma* sp. STP8 applied three times	A3	at seed sowing, at planting of seedlings, and 26 DAP
*Trichoderma* sp. STP8 application omitted Control	U	untreated plants from natural/untreated seeds

**Table 4:** Similarity of *rpb*2 sequences (%) between *Trichoderma* sp. strain STP8 and closely related species.

	*Trichoderma parahongkuii*^T^ (OR779476)	*Trichoderma hongkuii* ^T^ (OR779477)	*Trichoderma* sp. STP8 (PQ867587)	*Trichoderma neohongkuii* ^T^ (OR779481)
*Trichoderma parahongkuii* ^T^ (OR779476)		99.15	99.15	99.02
*Trichoderma hongkuii* ^T^ (OR779477)	99.15		100	99.63
*Trichoderma* sp. STP8 (PQ867587)	99.15	100		99.63
*Trichoderma neohongkuii* ^T^ (OR779481)	99.02	99.63	99.63	

The numbers in parentheses correspond to the GenBank accession numbers for each sequence. ^T^ denotes type (reference) culture, strain or voucher.

**Table 5:** Similarity of *tef*-1 sequences (%) between *Trichoderma* sp. strain STP8 and closely related species.

	*Trichoderma parahongkuii* ^T^ (OR779503)	*Trichoderma hongkuii* ^T^ (OR779504)	*Trichoderma neohongkuii* ^T^ (OR779508)	*Trichoderma* sp. STP8 (PQ867588)
*Trichoderma parahongkuii* ^T^ (OR779503)		96.65	96.28	96.76
*Trichoderma hongkuii* ^T^ (OR779504)	96.65		95.87	96.35
*Trichoderma neohongkuii* ^T^ (OR779508)	96.28	95.87		96.73
*Trichoderma* sp. STP8 (PQ867588)	96.76	96.35	96.73	

The numbers in parentheses correspond to the GenBank accession numbers for each sequence. ^T^ denotes type (reference) culture, strain or voucher.

**Table 6:** Effect of *Trichoderma* sp. strain STP8 on the phenotypic characteristics of lettuce in field growth.

Treatment	Head Weight (g)	Head Diameter (cm)	Leaf Length (cm)	Leaf Width (cm)
A1	530. 6 ab **^1^**	30. 3 bc	21. 4	19.5 a
B1	472. 9 ab	29. 1 cd	19. 9	17. 9 ab
C1	378. 2 c	26. 2 e	19. 0	15. 9 c
A2	535. 1 ab	33 a	21.9 a	19. 3 a
B2	567.2 a	32. 1 ab	21. 1	18. 2 ab
C2	544. 1 a	32. 5 a	21. 2	18. 5 ab
A3	562. 7 a	34 a	20. 5	19. 1 a
U	394. 3 c	28. 4 d	19. 2	17. 8 b
sD	24.7	0.6	0	0.6
LSD 5%	50.58	1.30	n.s.	1.3
LSD 1%	68.10	1.75	n.s.	1.75

^1^ Means with the same letter do not differ significantly at the *p* < 0.01, Duncan test. sD = standard deviation. LSD = Least significant difference, n.s. = Non-significant difference.

**Table 7:** Antagonism of *Trichoderma* sp. strain STP8 against some phytopathogenic fungi.

No.	Fungus	Average Colony Radius (mm)		
		Control ^1^	Test ^2^	I (%) ^3^
1.	*Alternaria solani*	78	16	79
2.	*Fusarium culmorum*	22	12	44
3.	*Fusarium solani*	75	19	75
4.	*Sclerotinia sclerotiorum*	90	21	77

^1^ Control = Axenic fungal culture + sterile mycelial plug instead of *Trichoderma* sp. STP8. ^2^ Test = Dual cultures *Trichoderma* sp. STP8 + named fungus. ^3^ I = Inhibition index.

## Data Availability

The original contributions presented in this study are included in the article. Further inquiries can be directed to the corresponding authors.
